# Transcranial Magnetic Stimulation in Tremor Syndromes: Pathophysiologic Insights and Therapeutic Role

**DOI:** 10.3389/fneur.2021.700026

**Published:** 2021-08-26

**Authors:** Jessica Frey, Christopher W. Hess, Liam Kugler, Manahil Wajid, Aparna Wagle Shukla

**Affiliations:** Department of Neurology, Fixel Institute for Neurological Diseases, University of Florida, Gainesville, FL, United States

**Keywords:** transcranial magnetic stimulation, tremor syndromes, essential tremor, dystonic tremor, Parkinson's disease, functional tremor, orthostatic tremor, theta burst stimulation

## Abstract

Transcranial magnetic stimulation (TMS) is a painless, non-invasive, and established brain stimulation technique to investigate human brain function. Over the last three decades, TMS has shed insight into the pathophysiology of many neurological disorders. Tremor is an involuntary, rhythmic oscillatory movement disorder commonly related to pathological oscillations propagated *via* the cerebello-thalamo-cortical pathway. Although tremor is the most common movement disorder and recent imaging studies have enhanced our understanding of the critical pathogenic networks, the underlying pathophysiology of different tremor syndromes is complex and still not fully understood. TMS has been used as a tool to further our understanding of tremor pathophysiology. In addition, repetitive TMS (rTMS) that can modulate brain functions through plasticity effects has been targeted to the tremor network to gain potential therapeutic benefits. However, evidence is available for only a few studies that included small patient samples with limited clinical follow-up. This review aims to discuss the role of TMS in advancing the pathophysiological understanding as well as emerging applications of rTMS for treating individual tremor syndromes. The review will focus on essential tremor, Parkinson's disease tremor, dystonic tremor syndrome, orthostatic tremor, and functional tremor.

## Introduction

Tremor is the most common movement disorder, defined as an “involuntary, rhythmic, oscillatory movement of a body part” (1). The phenomenology, pathophysiology, and treatment of the various tremor syndromes are highly nuanced and complex. Some features of tremor disorders may be difficult to distinguish from each other. Transcranial magnetic stimulation (TMS) is a painless and non-invasive technique used to study human brain function. TMS produces a magnetic field that induces a transient focal electric field in the targeted brain region. It can identify brain circuits involved in motor control and motor disorders and is an appealing technique for studying pathological tremors. It has shown promise as a potential treatment for tremors due to its ability to modulate the underlying pathological circuitry and brain functions. The current narrative review will discuss the role of TMS in understanding the pathophysiology and treatment for essential tremor (ET), Parkinson's disease (PD) tremor, dystonic tremor syndrome (DTS), and the less common or rare tremor syndromes such as orthostatic tremor (OT) and functional tremor.

## Pathophysiology of Tremor

Many models have been proposed to explain the pathophysiology of tremor. One important model relevant to all tremor syndromes is the oscillator hypothesis, which posits that a system can produce abnormal oscillatory activity under certain conditions that manifests clinically as tremor (2). There are four potential mechanisms that can lead to generation of these oscillations. These include mechanical properties of the body part, stretch reflexes in the extremity, oscillatory properties of neurons in certain brain regions, and oscillatory activity that occurs when feed forward or feedback systems involving the cerebellum become unstable (2, 3). With regards to the central oscillators, abnormal rhythmic activity generated within specific brain regions is propagated through networks critical for tremor; for instance, the cerebello-thalamo-cortical (CTC) network (2, 3). Brain regions with neurochemical disturbances are particularly susceptible to the generation of oscillations. For example, loss of cerebellar Purkinje cells in conjunction with GABAergic receptor abnormalities have been found to lead to tremor oscillations along the CTC pathway (4). Some studies have found loss of dopaminergic, serotonergic, and noradrenergic neurons in the brainstem lead to abnormal basal ganglia or thalamic oscillations (5).

Electromyography (EMG), electroencephalography (EEG), and neuroimaging such as functional magnetic resonance imaging (fMRI) are multiple pieces of the puzzle that have advanced our understanding of the brain circuitries and physiology involved in tremor syndromes. TMS is another important puzzle piece that has contributed to understanding the central mechanisms underlying the pathophysiology of tremor syndromes.

## TMS Techniques: Basic Concepts

**Figure 1:**
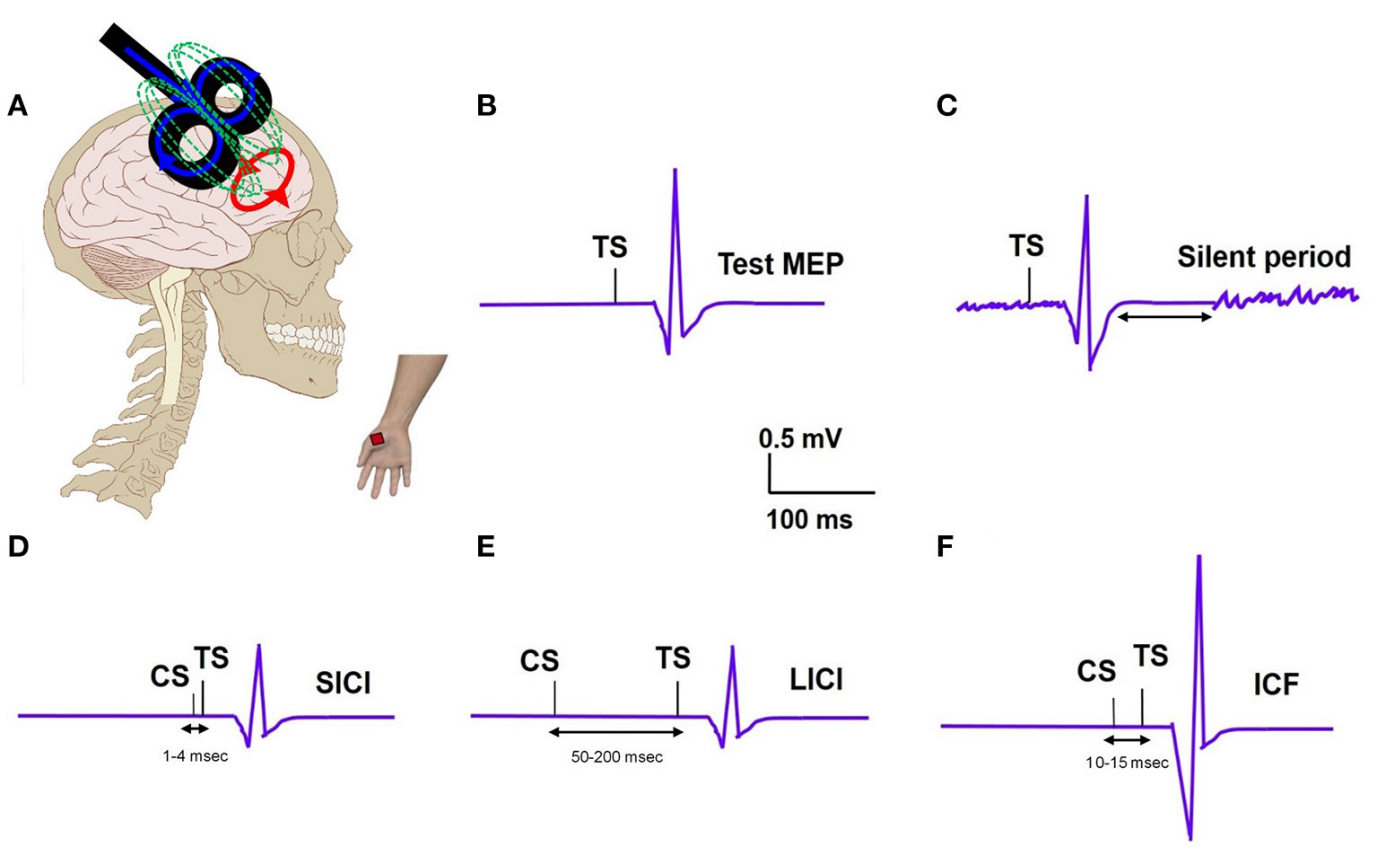
**(A)** TMS set up: TMS coil applied to the motor cortex and motor evoked potential (MEP) recording from the distal hand muscle; electric field (blue); magnetic field (green); induced electric field (red). **(B)** Test MEP: MEP elicited with a test stimulus (TS). **(C)** Silent period: Recording of EMG silence that occurs after the MEP when a suprathreshold TMS pulse is delivered during active muscle contraction. **(D)** Short interval intracortical inhibition (SICI); MEP elicited with TS is inhibited when preceded by a subthreshold conditioning stimulus (CS) at a short interval of 2–3ms. **(E)** Long interval intracortical inhibition (LICI); MEP elicited with TS is inhibited when preceded by a suprathreshold CS at a long interval (100ms). **(F)** Intracortical facilitation (ICF); MEP elicited with TS is increased when preceded by a subthreshold CS at an interval of 10–15 ms.

TMS examines brain circuitries by using a magnetic field to induce changes in neuronal excitability (Figure 1A). TMS includes single-pulse paradigms, paired-pulse paradigms, and repetitive-pulse paradigms. A single-pulse paradigm delivers a single pulse of TMS to specific brain regions in order to understand brain function. When a single pulse of TMS is delivered to the primary motor cortex (M1), this pulse subsequently generates a corresponding motor evoked potential (MEP) in the contralateral peripheral muscle, measured with an EMG recording (Figure 1B). MEP is a measure of corticospinal excitability. Single pulse TMS delivered during voluntary muscle contraction produces a period of EMG suppression known as the silent period (SP) (Figure 1C) (6). The SP evoked in the muscles of the upper limb originates largely from activation of cortical inhibitory interneurons with spinal contributions for the early part. SP is thought to represent motor cortex excitability involving the GABAergic receptors. When the SP is shortened, it reflects a dysfunctional inhibition. The resting motor threshold (RMT) is defined as the lowest stimulation intensity required to cause a muscle twitch in a target muscle for 5/10 pulses delivered (7). The active motor threshold (AMT), in contrast, is the motor threshold evoked by stimulation during a voluntary contraction of the peripheral muscle (7). These motor thresholds reflect the excitability of the motor cortex.

In paired-pulse paradigms, a conditioning stimulus (CS) is followed by a test stimulus (TS) with various interstimulus intervals (ISI) in order to generate MEPs that provide information about cortical excitability. The ratio of MEP amplitudes produced by a subthreshold CS and a suprathreshold TS when the ISI is short (1–4 ms) is known as short-interval intracortical inhibition (SICI) (Figure 1D). The ratio of MEP amplitudes produced by a suprathreshold CS and TS when the ISI is long (50–200 ms) is known as long-interval intracortical inhibition (LICI) (Figure 1E) (8). Intracortical facilitation (ICF) is an excitatory phenomenon whereby the MEP response is facilitated following a subthreshold CS paired with suprathreshold TS at an interstimulus interval of 10–15 ms (Figure 1F). A particular type of paired-pulse paradigm utilizes a CS targeted at the cerebellum and a TS at the motor cortex. When the ISI between these two pulses is 5–7 ms, the cerebellar cortex activated by the TMS pulse is observed to inhibit the contralateral motor cortex, a concept known as cerebellar-brain inhibition (CBI) (9). CBI paradigms can be used to study the cerebellar contribution, specifically involvement of the CTC pathway, in the pathophysiology of different tremor syndromes. In general, these paired pulse TMS paradigms can provide insights into the role of the motor cortex and the cerebellum, respectively, in tremor pathophysiology.

**Figure 2:**
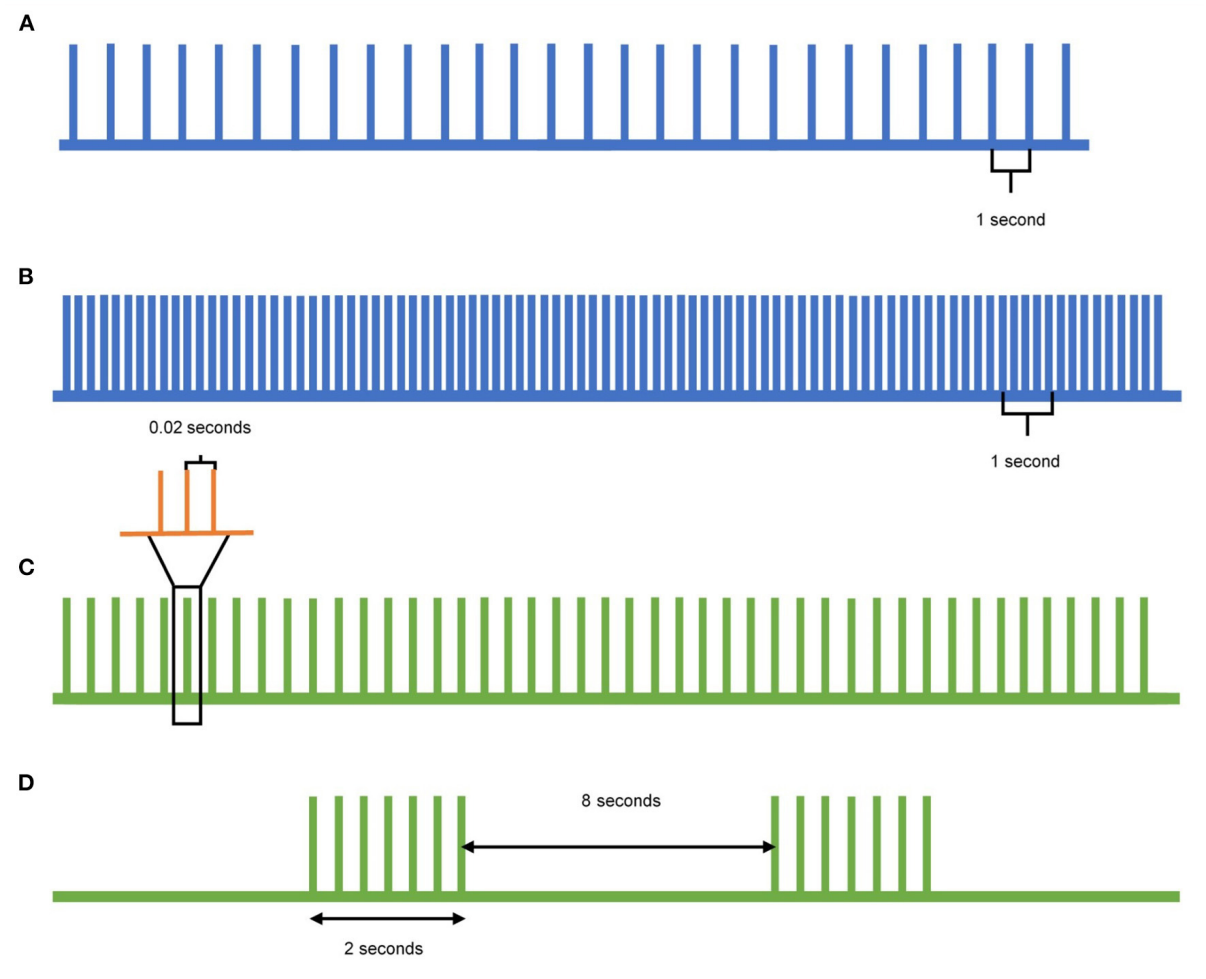
Pulse patterns of different rTMS protocols. **(A)** Low-frequency TMS is delivered at a rate of 1Hz or lower and acts as inhibitory stimulation. **(B)** High-frequency TMS is delivered at a rate of 5Hz or higher and acts as excitatory stimulation. **(C)** Continuous theta-burst stimulation (cTBS) delivers a triplet burst of pulses continuously, and acts as inhibitory stimulation. Each green pulse represents three pulses given at 50Hz (orange), whereas each green pulse is delivered at 5Hz. **(D)** Intermittent theta-burst stimulation (iTBS) delivers a triplet burst of pulses intermittently, acting as excitatory stimulation. Each triplet burst (green) is delivered at 5Hz over 2s with an 8s pause between.

In contrast to single- and paired-pulse TMS, which can detect changes in cortical excitability, repetitive TMS (rTMS) can be used to modulate the cortical excitability. When rTMS is delivered to specific cortical targets in the brain, specific aspects of brain activity can be influenced with the goal of translating these effects to clinical improvement (Figure 2). Low frequency (≤1Hz) rTMS mimics long-term depression, resulting in inhibitory effects in the cortex (Figure 2A). In contrast, high frequency (>5Hz) rTMS mimics long-term potentiation, resulting in excitatory changes (Figure 2B) (10). A specific type of rTMS known as theta-burst stimulation (TBS) uses triplet bursts of stimulation to deliver more pulses in a shorter time (3-pulse 50 Hz burst). When these triplet bursts are given continuously, known as cTBS, it exerts an inhibitory effect on the cortex similar to low frequency rTMS (Figure 2C). In contrast, when the triplet bursts are given intermittently, known as iTBS, it exerts an excitatory effect on the cortex similar to high frequency rTMS (Figure 2D) (9, 10). These neuromodulatory effects of rTMS, when targeted to the motor cortex and the cerebellum, can be leveraged to treat tremor syndromes. Following application of a rTMS paradigm, single- and paired-pulse TMS can detect differences in corticospinal excitability for clinical correlation.

A few studies have employed TMS techniques in healthy subject cohorts to understand tremor pathogenesis. Topka et al. examined the contributory role of the oscillations within the central brain circuits (11). rTMS delivered to the left motor cortex at a rate of 20 Hz, and an intensity of 120 % AMT was observed to transiently lead to the generation of tremor that correlated with an increase in frequency and stimulus intensity. In contrast, peripheral stimulation was unable to produce similar findings (11). The investigators concluded that the circuitry for tremor is mainly central. In another study with healthy subjects, a visuomotor task was used to induce an action tremor. When 6 Hz rTMS was applied to the M1, there was an increase in the action tremor, indicating that the modulation of tremors occurs centrally (11, 12).

In the following sections, we will discuss the role of TMS for each of the individual tremor syndromes. A summary of the TMS studies used to assess tremor pathophysiology (Table 1) and therapeutic role in individual tremor syndromes (Table 2) is provided.

**Table 1:** TMS studies for understanding the pathophysiology of tremor syndromes.

**References**	**Participants**	**TMS protocol**	**Results**
Britton et al. (13)	10 PD vs. 12 ET vs. 10 HC	Single pulse TMS over M1 at 110% RMT	Tremor reset occurred for both ET and PD groups; latency to tremor return was prolonged, period of tremor was shortened in PD compared to ET or HC
Pascual-Leone et al. (14)	9 ET vs. 12 PD (postural tremor)	Single and paired pulse TMS over M1	Tremor reset occurred equally for both ET and PD groups and correlated with stimulus intensity and duration of SP; tremor reset bilaterally even with unilateral stimulation
Mills and Nithi (15)	5 OT	Single pulse TMS over the contralateral leg motor cortex while patients were standing	OT was not reset by cortical stimulation
Tsai et al. (16)	2 OT	Single pulse TMS over the contralateral leg motor cortex at 110% RMT while patients were standing	OT reset by cortical stimulation
Romeo et al. (17)	10 ET vs. 8 HC	Single and paired pulse TMS over M1 with ISIs of 3, 5, 20, 100, 150, and 200 ms; stimulation delivered at 80% RMT for short ISI and 150% for long ISI	No significant difference in RMT, SP, or SICI between ET and HC
Manto et al. (18)	3 OT with pancerebellar atrophy	Single pulse TMS over the contralateral leg motor cortex at 120% RMT with delays from time of EMG recording of the quadriceps femoris to TMS pulse delivery ranging from 25 to 60% while patients were standing	OT reset by motor cortex stimulation, which may suggest primary OT and OT associated with cerebellar atrophy have distinct pathophysiological mechanisms
Wu et al. (19)	6 OT	Single pulse TMS over the contralateral arm motor cortex while patients were standing	OT was not reset by motor cortex stimulation
Pinto et al. (20)	9 ET vs. 10 HC; medications discontinued 24 h prior to study	Conditioning stimulus delivered to right cerebellum and test stimulus delivered to left motor cortex with ISI values of 3, 9, and 15 ms	No significant difference in MEP or CBI between ET and HC; tremor was reset with stimulation over M1 but not over the cerebellum
Shukla et al. (21)	24 ET vs. 24 HC	Single pulse TMS over M1	No significant difference in SP between ET and HC
Spiegel et al. (22)	7 OT	Single pulse TMS over contralateral and ipsilateral cortical leg motor cortex at 110% RMT vs. lumbar magnetic stimulation vs. peripheral nerve stimulation	Tremor was reset in the bilateral legs with unilateral cortical stimulation, but was not reset with lumbar or peripheral nerve stimulation
Molnar et al. (23)	7 ET with DBS of the VIM in the dominant hemisphere vs. 11 HC with TMS; not on medications at time of study	Single pulse TMS over M1 at 100–150% of RMT; DBS conditions included ON, HALF, and OFF stimulation	No difference in SICI, LICI, or active ICF between ET and HC; significantly higher MEP with DBS ON compared to HC at high but not low TMS intensity, suggesting VIM DBS activates the target area
Lo et al. (24)	20 ET vs. 20 HC; not on medications at time of study	Single pulse TMS over M1 at 110% RMT in 3 s intervals at rest and during a motor imagery task 2 s before the TMS pulse	MEP amplitude increased following motor imagery in HC, but not in ET; no significant difference in RMT between ET and HC at baseline; no correlation between motor imagery scores and ET frequency or severity
Mazzochio et al. (25)	10 PD vs.16 ET vs.10 HC vs. 8 PT; not on medication prior to the study	Single-pulse TMS over the M1 combined with changes in shoulder position to influence motor cortical outflow	MEP amplitude decreased in HC and ET under resting conditions but increased under active conditions; no difference in MEP amplitude in PD at rest but decreased during activation
Avanzino et al. (26)	15 ET vs. 11 HC; medication stopped 72 h before study	Single session of 600 pulses of 1 Hz rTMS over the right lateral cerebellum at 90% RMT	At baseline, patients with ET had longer touch duration and temporal variability of movement compared to HC; following inhibitory rTMS, these parameters normalized
Ni et al. (27)	10 PD OFF medication vs. 10 HC	CBI paired pulse paradigm consisting of TMS pulse to the cerebellum followed by TMS pulse to the M1 with ISI ranging from 3 to 8 ms; CBI was tested at rest and with arm extension	Rest tremor reset by M1 but not cerebellar stimulation; postural tremor reset by both M1 and cerebellar stimulation; CBI abnormal in both rest and postural tremor and correlated with the degree of reset caused by cerebellar stimulation
Rogasch et al. (12)	26 HC with action tremor induced by a visuomotor task	Single session o 600 pulses of 6 Hz rTMS over the M1 at 80% AMTActive vs. sham	Peak power and tremor frequency increased following active rTMS; decreased corticospinal excitability but increased amplitude following active rTMS
Chuang et al. (28)	13 ET vs. 18 HC	Single pulse TMS to the M1; Paired-pulse TMS with subthreshold conditioning stimulus 80% AMT and test pulse at 100% AMT at ISIs of 3 and 12 ms; 600 triplet bursts of cTBS targeted at either the M1 or premotor cortex at 80% AMT	No change in SICI or SP in ET but reduced SICI and prolonged SP following cTBS in HC; reduction in MEP amplitude in both ET and HC, but sustained longer in HC
Lu et al. (29)	10 PD vs. 10 ET; medication discontinued 24 h prior to study	Single pulse TMS over the M1; paired pulse TMS over M1, SMA, and cerebellum in random order	Tremor reset occurred for both M1 and SMA targets in both groups; tremor reset index was significantly higher for M1 as compared to SMA stimulation in PD group, but no difference in ET group; no tremor reset with cerebellar stimulation; no significant difference in MEP, LICI, or SP between PD or ET group
Hanajima et al. (30)	18 ET vs. 19 HC; medications stopped 18 h before study	CBI paradigm consisting of conditioning stimulus with insentiy at 95% of AMT directed at the cerebellum and test stimulus applied over the motor cortex with ISIs of 4, 5, 6, 7, 8, and 10 ms	Abnormal CBI in ET compared to HC
Pattamon et al. (31)	12 ET vs. 8 DT	CBI paradigm consisting of a conditioning stimulus delivered to the cerebellar cortex and a test stimulus delivered to the contralateral motor cortex with an ISI of 5 ms	M1 stimulation reset both ET and DT whereas cerebellar stimulation reset ET more so than DT
Khedr et al. (32)	21 ET vs. 20 HC; no medications 1 week before study	Single pulse TMS over M1 at intensities ranging from 110 to 150% RMT and AMT	RMT and AMT were significantly decreased compared to HC; no difference in SP between ET and HC
Panyakaew et al. (33)	21 ET vs. 22 DTS vs. 19 HC; not on medications prior to study	CBI paradigm consisting of a conditioning stimulus delivered to the cerebellar cortex and a test stimulus delivered to the contralateral motor cortex with an ISI of 5 ms	Correlation between CBI and tremor severity scale only in ET; CBI significantly reduced in DT but not in TAWD compared to ET or HC
Leodori et al. (34)	10 PD in the OFF medication state	Single-pulse TMS over M1 at 80% AMT	Both rest and re-emergent tremor were reset following stimulation of M1
Helmich et al. (35)	14 PD (rest and re-emergent tremor)	Single-pulse TMS over M1 and cerebellum	Both rest and re-emergent tremor were reset following stimulation of M1 but only re-emergent tremor was reset following stimulation of the cerebellum

*AMT, active motor threshold; CBI, cerebellar-brain inhibition; CSP, cortical silent period; cTBS, continuous theta burst stimulation; DT, dystonic tremor; DTS, dystonic tremor syndrome; ET, essential tremor; HC, healthy controls; ICF, intracortical facilitation; LICI, long intracortical inhibition; M1, primary motor cortex; MEP, motor evoked potential; PD, Parkinson's disease; PT, physiologic tremor; RMT, resting motor threshold; rTMS, repetitive transcranial magnetic stimulation; SICI, short intracortical inhibition; SMA, supplementary motor area; TAWD, tremor associated with dystonia; TMS, transcranial magnetic stimulation*.

**Table 2:** rTMS studies for therapeutic use in tremor syndromes.

**References**	**Participants**	**Target**	**Stimulation parameters and study design**	**Number of pulses per session**	**Duration**	**Results**
**Essential tremor**						
Gironell et al. (36)	10 ET; patients were allowed to continue medications during the study	Posterior cerebellum (2 cm inferior to the inion)	Thiry 10-s trains of 1 Hz rTMS at 100% RMT with 30 second intertrain intervals Crossover design of active and sham rTMS separated by 1 week	300	Single session	Significant decrease in tremor rating scale and improvement in accelerometry scores 5 min following active rTMS compared to sham rTMS, but no difference at 60 min after stimulation
Avanzino et al. (26)	15 ET and 11 HC; medications stopped at least 72 h before study	Right lateral cerebellum (3 cm lateral and 1 cm inferior to the inion)	One 10-min train of 1 Hz rTMS at 90% RMT	600	Single session	No change in frequency or intensity of tremor by clinical rating scales
Hellriegel et al. (37)	10 ET and 10 HC; patients were allowed to continue antitremor medications if started at least 4 weeks before the study	M1	Two 20-s trains of 50-Hz cTBS at 80% AMT with an intertrain interval of 60 s crossover design of active and sham rTMS separated by at least 1 week	600	Single session	Significant reduction in tremor as measured by accelerometry 45 min following active stimulation as compared to sham; no significant difference in the tremor rating scale or MEP amplitude
Popa et al. (38)	11 ET and 11 HC; patients were allowed to continue antitremor medication	Cerebellum (repeated over lobule VIII of each cerebellar hemisphere)	One 15-min train of 1 Hz rTMS at 90% RMT to each cerebellar hemisphere open label study	1,800 pulses per session (9,000 pulses total)	5 days	Significant reduction in tremor rating scale and improvement in tremor amplitude by accelerometry with sustained response up to 3 weeks; restoration of functional connectivity in the CTC network to a near normal level following stimulation
Chuang et al. (28)	13 ET and 18 HC	M1 or PMC	One 40-s train of 50-Hz cTBS at 80% AMT Crossover design of active vs. sham cTBS separated by at least 1 week	600	Single session	Significant reduction in tremor amplitude but no change in tremor frequency; no difference between motor vs. PMC
Bologna et al. (39)	16 ET and 11 HC	Cerebellum (right cerebellar hemisphere)	One 40-s train of 50 Hz cTBS at 80% AMT crossover design of active and sham cTBS separated by at least one week	600	Single session	No significant change in tremor rating scale or kinematic analysis of tremor following active stimulation; reduction of MEPs in the HC and not the ET patients, suggesting dysfunction of the CTC connectivity in patients with ET.
Badran et al. (40)	10 ET	Pre-SMA	20 min of 1 Hz rTMS at 110% RMT Randomized to active vs. sham	1,200 per session (18,000 pulses total)	15 days	Significant reduction in the tremor rating scale compared to baseline in both groups but sustained reduction at 4 and 8 week follow-up persisted only in the active group.
Shin et al. (41)	22 ET; patients were allowed to continue antitremor medications during study	Cerebellum (each cerebellar hemisphere, 3 cm lateral and 1 cm inferior to the in inon)	Twenty 30-s trains of 1 Hz rTMS with a 10 s intertrain interval at 90% RMT Randomized to active vs. sham	1,200 per session (6,000 pulses total)	5 days	No significant difference in tremor rating scale (immediately after rTMS: 33% reduction in active vs. 20% reduction in sham; 4 weeks following rTMS: 31% reduction in active vs. 17% reduction in sham)
Olfati et al. (42)	23 ET	Cerebellum (right then left cerebellar hemisphere, 1/3 distance from the inion to the mastoid process)	Two 15-min trains of 1-Hz rTMS at 90% RMT with a 5-min intertrain interval Crossover design of active vs. sham with a 2-month washout period	1,800 pulses per session (9,000 pulses total)	5 days	Significant reduction in tremor rating scale following active or sham rTMS but no significant differences between active or sham rTMS
**Parkinson's disease tremor**						
Bologna et al. (39)	13 PD and 10 HC; patients discontinued antitremor medications the night before the study	Cerebellum (in the hemisphere ipsilateral to the tremulous side of the body, 3 cm lateral and 1 cm inferior to the inion)	One 40-s train of 50-Hz cTBS at 80% AMT Crossover design for active vs. sham during off medication state at least 1 week apart	600	Single session	No significant difference in tremor amplitude or frequency between active and sham stimulation by kinematic analysis; significant reduction in M1 excitability following active but not sham stimulation
Lefaivre et al. (43)	50 PD; patients continued antitremor medication during the study	Cerebellum (medial cerebellum defined as directly beneath the inion, or lateral cerebellum, defined as 3 cm lateral and 1 cm inferior to the inion)	One 15-mi train of 1 Hz rTMS at 120% RMT Active (medial or lateral) vs. sham during on medication state	900	Single session	Significant improvement in rest tremor rating score by Kinesia motion sensor following medial and lateral cerebellar stimulation compared to sham
Fricke et al. (44)	20 PD; patients discontinued antitremor medications the night before the study	M1 and dPMC	Forty 25-s trains of 1-Hz ADS-rTMS at 95% RMT with a 5-s intertrain interval Crossover design for active and sham during off medication state at least 1 week apart	1,000 (pairs of stimuli)	Single session	No significant difference in UPDRS, finger tapping, or tremor by kinematic analysis between active and sham stimulation
**Dystonic tremor syndrome**						
Murase et al. (45)	9 WC; one patient with tremor	M1, PMC, SMA	One 21-min train of 0.2 Hz rTMS at 85% RMT Crossover design to different rTMS target sites and sham; each target site separated by at least 1 week	250 pulses per session (750 pulses total)	single session per target site	Significant improvement in handwriting scores with PMC stimulation; no comment on tremor
Huang et al. (46)	18 WC; one patient listed as having tremor	dPMC	One 40-s train of 50-Hz cTBS at 80% AMT Randomized to active or sham stimulation	600 pulses per session (3,000 pulses total)	5 days	Subjective improvement in writing following active rTMS but no significant difference in writing speed or spiral between groups; no comment on tremor
Kimberley et al. (47)	17 FHD; 2 patients listed as having tremor	dPMC	One 30-min train of 1-Hz rTMS at 90% RMT Crossover design between active and sham rTMS separated by 10 days	1,800 pulses per session (9,000 pulses total)	5 days	No significant difference in clinical measures between active and sham stimulation; no comment on tremor
Pirio Richardson et al. (48)	8 CD; 3 listed as having dystonic tremor	ACC, dPMC, M1, SMA	One 15-min trains of 0.2-Hz rTMS at 85% RMT Crossover design to different rTMS target sites and sham with 2 day washout	180 pulses per target (540 pulses total)	single session per target site	Trend for improvement in TWSTRS score for the dPMC and M1 sites; no comment on tremor
de Oliveira Souza et al. (49)	Case report: 1 patient with FHD and associated tremor	PMC	One 20-min train of 1 Hz rTMS at 80% RMT	1,200 pulses per session (18,000 pulses total)	15 days	Significant improvement following stimulation but benefits not sustained at 3 month follow-up; no specific comment on tremor
**Orthostatic tremor**						
Gallea et al. (50)	9 OT	Cerebellum (over lobule VIII of each cerebellar hemisphere)	Two 15-min trains of 1 Hz rTMS at 90% RMT over each cerebellar hemisphere Open label design	1,800 pulses per session (9,000 pulses total)	5 days	No significant difference in FABRS or standing duration following rTMS; significant reduction in tremor amplitude by EMG analysis following rTMS; functional connectivity between lateral cerebellum and SMA which was abnormally increased in patients with OT compared with HC was reduced following stimulation
Hu et al. (51)	10 OT; 9 HC; patients discontinued antitremor medication at least 12 h before study	Cerebellum (3 cm lateral to the inion on the line joining the inion and the external auditory meatus)	One 15-min train of 1 Hz rTMS at 90% RMT Crossover design for active and sham rTMS separated by 1 day	900	Single session	Significant improvement in FABRS and standing duration immediately following active rTMS as compared to sham rTMS, but no sustained difference 1 h after rTMS; CBI significant increased at baseline compared to HC and normalized following active rTMS
**Functional tremor**						
Dafotakis et al. (52)	11 FT	M1 (contralateral to the affected hand)	30 total pulses of 0.2 Hz rTMS at 120% (15 pulses) and 140% (15 pulses) of the RMT Open label study	30	Single session	Using kinematic motion analysis, there was a significant reduction in tremor following rTMS, with a sustained response in about half of patients
Taib et al. (53)	18 FT	M1 (contralateral to the affected limbs)	800 × 2 biphasic pulses of 1 Hz rTMS at 90% RMT Randomized to active vs. sham followed by an open-label phase in combination with hypnosis	800 pulses per session (4,000 pulses total during randomized phase)	Randomized phase: 1 session for 5 consecutive days; open-label phase: 1 session weekly for 3 consecutive weeks	Significant decrease in PMDRS following active rTMS with sustained benefit at 6 month follow-up and throughout the open-label phase

*ACC, anterior cingulate cortex; ADS-rTMS, associative dual-site repetitive transcranial magnetic stimulation; AMT, active motor threshold; CBI, cerebellar-brain inhibition; CD, cervical dystonia; CSP, cortical silent period; cTBS, continuous theta burst stimulation; CTC, cerebello-thalamo-cortical; DT, dystonic tremor; dPMC, dorsal premotor cortex; DTS, dystonic tremor syndrome; ET, essential tremor; FABRS, Fullerton advanced balance rating scale; FHD, focal hand dystonia; FT, Functional tremor; HC, healthy controls; M1, primary motor cortex; MEP, motor evoked potential; OT, orthostatic tremor; PD, Parkinson's disease; PMC, premotor cortex; PMDRS, psychogenic movement disorder rating scale; RMT, resting motor threshold; rTMS, repetitive transcranial magnetic stimulation; SICI, short intracortical inhibition; SMA, supplementary motor area; TAWD, tremor associated with dystonia; TMS, transcranial magnetic stimulation; TWSTRS, Toronto western spasmodic torticollis rating scale; UPDRS, unified parkinson's disease rating scale; VIM, ventral intermediate nucleus of the thalamus; WC, writer's cramp*.

## Essential Tremor

ET is the most common tremor syndrome, occurring in 4% of adults over the age of 40 years (54, 55). The clinical manifestation of ET typically includes a combination of postural and action tremors of the arms. In some patients the head, voice, legs, and trunk may also be involved (56). Propranolol and primidone are mainstay pharmacological therapies; however, many patients discontinue medical treatments given an average of 50% improvement in symptoms and a relatively high incidence of medication-related side effects. Surgical techniques, including deep brain stimulation (DBS) and focused ultrasound, can be considered in severe, medication-refractory cases (54), but they have limitations such as side effects and costs. Therefore, rTMS in ET has gained interest as a potential alternative option for treating tremor.

ET is generally accepted to result from pathologic oscillations within the CTC pathway. Prior kinematic studies have demonstrated that rhythmic finger movements in patients with ET had higher variability than healthy controls, supporting cerebellar dysfunction as an underlying factor (26). Lesions in the cerebellum and the motor cortex have been observed to sometimes lead to the disappearance of symptoms (2, 3). Imaging studies have shown increased activity in the cerebellum and the motor cortex (57). Some pathological studies have found degenerative changes in the cerebellum; for example, the loss of Purkinje cells and focal axonal swelling that likely leads to abnormal GABAergic output and generation of pathological oscillations (55). Despite a growing understanding of the CTC pathway's involvement, whether the main tremor oscillator resides in the cerebellar cortex or is further downstream in the thalamus or motor cortex, and whether cerebellar involvement is related to decoupling remains an important physiologic question (5).

### Pathophysiological Insights From TMS

Except for one study that found decreased RMT and AMT (32), the vast majority of studies have demonstrated that the baseline excitability in patients with ET is not significantly different from matched, healthy controls (17, 20, 21, 23, 24, 28). Resetting tremor with a TMS pulse applied to the cortical brain has further facilitated understanding of the pathophysiology. Resetting studies assume that if the tremor rhythm is disrupted or reset by the TMS pulse, the area stimulated must be involved in the tremor circuit (58). A single pulse of TMS targeted at the motor cortex was observed to reset tremor in ET (13, 14, 20, 29), however, it somewhat surprisingly did not reset when delivered to the cerebellum (20, 29). The authors speculated that the distal thalamo-cortical part of the CTC pathway might have a more prominent contribution to tremor generation than the proximal cerebello-thalamic part, which is why a single pulse of TMS to the primary motor cortex reset ET, but the cerebellum did not (29). Paired-pulse TMS studies investigating CBI have found variable results, with one study demonstrating no difference in CBI (20), and another study demonstrating reduced CBI in ET compared to healthy controls (30). The precise target within the cerebellum and the number of study participants differed between the two studies, which may be why there was a difference in their results (58).

TMS studies have also investigated the role of the cerebellum in ET generation by implementing an inhibitory cTBS protocol directed at the cerebellum. In one study, there was normalization of touch duration and temporal variability of ET with the cTBS protocol (26). In another study, cTBS targeted at the cerebellum led to a reduction in MEP amplitude in healthy controls, which could not be replicated in patients with ET. The authors interpreted this lack of response observed in the ET group to indicate dysfunction of the CTC pathway (39). Similarly, when inhibitory rTMS was targeted to the motor cortex, there was evidence of prolonged SP and reduced SICI in healthy controls, but no changes in ET, suggesting impaired plasticity and less modifiable motor cortical circuits (28).

### Therapeutic Use of rTMS

Since the thalamus in the CTC pathway is too deep to reach with conventional TMS pulses, the cerebellum and the motor cortex remain the two best potential candidates for clinical efficacy. Most studies to date have targeted the cerebellum (58). In one study, low frequency rTMS to the cerebellum led to a significant decrease in clinical tremors immediately following the stimulation, correlating with accelerometer-based tremor improvement (36). However, this improvement did not persist 1 h following stimulation, which is likely attributable to a single stimulation session. Another study, which used a single session cTBS protocol to the cerebellum, did not demonstrate improvements measured clinically or with kinematic analysis (26, 39). Further studies expanded the number of total pulses provided to patients by repeating stimulation sessions over several days. An open-label low frequency paradigm that extended the number of sessions to 5 consecutive days found significant improvement in the clinical rating scale and tremor as measured by accelerometry (38). These improvements lasted up to 3 weeks following stimulation (38). This study also demonstrated restoration of CTC connectivity on the fMRI (38). However, two other low frequency rTMS studies with a similar 5-day paradigm found no significant difference between active and sham stimulation conditions (41, 42).

In addition to the cerebellum as a target, the M1, the premotor cortex (PMC), and the supplementary motor area (SMA) have been pursued as potential rTMS targets for the treatment of ET. In one study, 600 triplet bursts of cTBS to the M1 or PMC led to significant reduction in tremor amplitude with no change in tremor frequency (28). In another study with cTBS targeted at the M1, there was reduction of tremor measured with accelerometer studies; however there was no significant change in the clinical tremor rating scale, which may be due to the implementation of only a single stimulation session (37). Inhibitory low frequency rTMS was pursued in one study for 15 stimulation days and the investigators chose the pre-SMA as the target. The study found that compared to sham stimulation, there were sustained benefits at 8-week follow-up in the active stimulation group (40).

While multiple brain targets have been pursued, albeit with limited data, a recent meta-analysis evaluating non-invasive brain stimulation for ET found that there was tremor improvement regardless of the tremor rating scale used, the stimulation site, the number of sessions, or how long after stimulation outcome measures were assessed (54). However, based on methodological merits including randomization, blinding, inclusion of sham-control, and duration of benefits, the overall evidence was deemed to be of moderate quality. Thus, studies involving multiple sessions and larger samples are needed to further clarify the role of rTMS in ET.

## Parkinson's Disease Tremor

PD is a neurodegenerative disorder characterized by motor symptoms including tremor, rigidity, bradykinesia, and postural instability (8). The most classical type of PD tremor is rest tremor, which is commonly asymmetric and/or unilateral at the time of onset (1). Rest tremor is defined as a tremor that occurs in a body part that is not voluntarily activated and is completely supported against gravity (ideally, resting on a couch). During postural elevation of arms, rest tremor typically subsides for a transient period, followed by delayed re-emergence, which is known as re-emergent tremor (1). In addition to rest and re-emergent tremors, some patients may also have a postural tremor, which is typically a higher frequency than rest tremor (59). In some circumstances, it can be difficult to clinically distinguish rest, re-emergent, and postural tremor. Rest tremor is commonly treated with dopaminergic and anticholinergic medications. In medication-refractory cases, DBS of the subthalamic nucleus or globus pallidus internus can be considered (8).

The underlying pathophysiology of PD tremor is complex and not fully understood. Tremor-predominant PD has more pronounced degeneration of the medial substantia nigra compared to akinetic-rigid PD (3). There is evidence to support both the basal ganglia projecting to the motor cortex and the CTC pathway as possible primary oscillators for PD tremor (60). Neuroimaging studies have shown dopaminergic deficits primarily contributing to rest tremor (3). Recently a “dimmer-switch” model was proposed that posits that the basal ganglia activates the tremor (“light switch”), whereas the CTC pathway modulates the tremor amplitude (“light dimmer”) (60). This model helps to explain a paradox that unlike the other motor symptoms, PD tremor does not necessarily correlate with the degree of basal ganglia disease (60). In addition, this model also provides a potential explanation for the varying responses of PD tremor to dopamine. Dopamine-resistant PD tremor may have a larger contribution from the cerebellum, whereas dopamine-responsive tremor may have a larger contribution from the thalamus or globus pallidus internus (5).

### Pathophysiological Insights From TMS

Single-pulse TMS delivered to the M1 was found to reset the rest component (13, 14, 27, 29), whereas a single-pulse over the cerebellum reset the postural component of the PD tremor (27, 29). In one study, application of the cerebellar pulse reset the re-emergent subtype of postural tremor suggesting that the cerebellum is involved in the oscillatory mechanism controlling pure postural and re-emergent postural tremor (35). Ni et al. found that rest tremor was reset with M1 stimulation; however, postural tremor was reset by both M1 and cerebellar stimulation (27). Ni et al. also found that compared to the healthy controls, CBI was reduced in PD tremor, which correlated with the degree of postural tremor reset caused by the cerebellar stimulation (27). These findings imply that the motor cortex may have more consistent involvement in the pathogenesis of rest tremor whereas the cerebellum likely contributes to pure postural and re-emergent subtypes of postural tremor.

### Therapeutic Use of rTMS

A multitude of studies have demonstrated motor symptom improvements with rTMS in PD. These studies employing either low or high frequency protocols have targeted the M1, SMA, and dorsolateral prefrontal cortex (8, 61–63). However, there is a paucity of data for PD tremor benefits directly related to rTMS. Bologna et al. used a cTBS protocol targeted at the cerebellum and found that motor cortex excitability was reduced following active stimulation, but there was no change in rest tremor assessed clinically or with kinematic analysis (64). The study authors concluded that the CTC pathway was not primarily driving the rest tremor. However, in another study by Lefaivre et al., rest tremor as rated by kinematic parameters was reduced by low-frequency rTMS targeted to the medial and lateral cerebellum (43). These two studies used different stimulation techniques and there were differences in clinical populations, which could explain the conflicting results. For example, Bologna et al. focused on rest tremor and evaluated tremor during the off-medication state, whereas Lefaivre et al. included patients with tremor-predominant and akinetic-rigid PD, and all assessments were performed during the on-medication state (43, 64).

A novel protocol known as associative dual-site rTMS was implemented by Fricke et al., who hypothesized that simultaneous targeting of the dorsal premotor cortex and the M1 in a coordinated fashion might lead to decoupling of pathogenic oscillatory tremor activity (44). However, the study found no clinical improvements, suggesting that the optimal target site for PD tremor is still not clear (44). Based on the data from pathophysiological studies, it is reasonable to postulate that the rest and postural tremors are likely amenable to different stimulation sites. More extensive studies involving multiple targets and multiple stimulation sessions will further clarify the role of rTMS in PD.

## Dystonic Tremor Syndrome

Tremor is a part and parcel feature of dystonia. When the tremor is found in a body part affected by dystonia, it is labeled as dystonic tremor (DT) (1). On the other hand, if dystonia and tremor are seen in different body parts, it is referred to as tremor associated with dystonia (TAWD) (1). Prevalence rates for tremors in dystonia are higher when patients are diagnosed with adult-onset focal dystonia and in dystonia that begins to spread from the original affected body part (65). In most patients, tremor manifests during posture or voluntary movements, but some patients may have tremor at rest (65). Few studies in the literature have specifically addressed DTS treatment, with most being retrospective and non-randomized studies (66). The available literature has not found consistent improvements with oral pharmacological therapies; however the use of botulinum toxin injection therapy is promising (66). There is also evidence to support that medication-refractory DTS responds to DBS targeted to the globus pallidus internus or the thalamus (66, 67).

The pathophysiology of DTS is not well-characterized (2). Neuroimaging studies have demonstrated that both the cerebellum and connections to the basal ganglia are involved (5). In a recent functional MRI study, task-based connectivity of the cerebellum, globus pallidus internus and motor cortex was significantly more affected in DT than ET (57). It is unclear whether the oscillators within the CTC pathway or the basal ganglia projections are the primary drivers for DTS (5). Furthermore, DT and dystonia may have distinct pathophysiological substrates as they may respond to different medical and surgical treatments (68). For example, DT may respond to medications such as propranolol and primidone that are not usually employed for dystonia, and although DBS is typically targeted to the globus pallidus internus for dystonia patients, the thalamus may be a viable option for DT.

### Pathophysiological Insights From TMS

Single-pulse TMS studies have demonstrated DT could be reset with stimulation over the motor cortex as well as the cerebellum (31). However, when comparing DT with ET, stimulation over the cerebellum was observed to have more robust effects. The role of the cerebellum was further explored in a follow-up study that used a CBI paradigm to distinguish the characteristics of DT from TAWD (33). CBI was reduced in DT but not in TAWD, indicating less inhibition in the CTC pathway (33). Compared to TAWD and ET, DT had higher variability and increased instability. During motor task (especially complex tasks) performance, DT became more unstable likely due to abnormal interactions of the motor command with the central oscillator (33). The study also found that the characteristics of TAWD were closer to ET than DT.

### Therapeutic Use of rTMS

There have been numerous studies evaluating rTMS for therapeutic benefit in dystonia. The rTMS studies that have assessed dystonia have tried to alleviate symptoms in focal hand dystonia (45–47, 69–75), cervical dystonia (48, 76, 77), blepharospasm (78–80), and generalized dystonia (81–83). However, these studies have not focused on DTS in particular. Only five studies reported inclusion of patients with DTS as part of the baseline characteristics. However, the tremor outcomes were not separately analyzed, making it difficult to draw conclusions about rTMS specifically in DTS (45, 47–49, 73). Future studies should include separate cohorts of DT and TAWD and compare DTS with dystonia in general to elucidate the therapeutic role of rTMS in this patient population.

## Orthostatic Tremor

OT is a rare disorder characterized by a high frequency (13–18 Hz) tremor recorded during EMG from the leg muscles (1). OT results in unsteadiness when standing, which improves with walking or sitting (1). OT is defined as primary when the tremor is the sole manifestation with no additional neurological features. “OT plus” refers to tremor in combination with other associated neurological features such as an ET-like arm tremor or parkinsonian features (84). Most OT cases are idiopathic; some patients reportedly have cerebellar degeneration, paraneoplastic syndromes, or other metabolic disturbances that may be contributory (85). Since OT is rare, evidence to support treatment is limited and also challenging to study systematically. The most commonly used medication is clonazepam, as it can moderately reduce tremors in about one-third of patients and may eliminate symptoms in some patients (85). Beta-blockers and anticonvulsants are other medications that have shown mild benefits in a small percentage of patients (86). Some studies have reported that DBS targeted to the thalamus is effective, but this requires further study (87–89).

OT is a unique tremor syndrome for many reasons: tremor is only induced in weight-bearing positions, frequency is high (≥13Hz) compared to frequencies of other pathological tremors (4–12Hz) (3), and high coherence is observed between EMG signals recorded from muscles in the legs, arms, and face (90). Unlike the other tremor syndromes, the coherence pattern recorded from homologous muscles in both sides of the body does not change over extended periods. The oscillator for OT likely resides in the posterior fossa, most likely the cerebellum and its connections with the brainstem and spinal motor neurons (3, 85, 91).

### Pathophysiological Insights From TMS

A few studies have used single-pulse TMS techniques with variable results. While some studies were unable to reset OT (15, 19), others targeting the leg area in the motor cortex found significant effects (16, 18, 22, 92), thus supporting the hypothesis of a supraspinal generator for the tremor (16, 22, 85). Evidence suggests that OT may be modulated along the CTC pathway and downstream to the spinal cord from the central tremor generator (88). In a recent study, CBI was found to be significantly increased in the OT group compared to healthy controls, further supporting the involvement of the cerebellum in the pathophysiology of OT (50).

### Therapeutic Use of rTMS

Only two small clinical trials have studied rTMS for OT. Both studies targeted the cerebellum and used the same paradigm of 900 pulses of 1 Hz rTMS at 90% of the RMT (50, 51). While Gallea et al. employed multiple stimulation sessions, Hu et al. used a single session of active vs. sham in a crossover design (50, 51). Gallea et al. found no improvements in clinical assessment, but the accelerometer analysis revealed a decrease in tremor amplitude sustained up to 3 weeks (50). There was also a decrease in functional connectivity between the lateral cerebellum and SMA (50). Hu et al. found improvements in standing time with active stimulation that correlated with changes in CBI (51). Future randomized studies should employ large samples with multiple sessions to determine if these clinical improvements persist.

## Functional Tremor

Up to 20% of patients presenting to the movement disorder clinics are ultimately diagnosed with a functional movement disorder (FMD), which refer to various movement symptoms that are unexplained by organic disease or have features that are only partially explained by underlying organic disease (93). Recent studies have found a clear interplay between neurological and psychological components (53, 93). Treatment of FMDs is quite challenging, and patients may experience significant impairment in their quality of life. An integrated and transparent approach involving multiple disciplines is most helpful. Cognitive-behavioral therapy is a promising treatment option that helps identify how the thought processes may affect emotions or behaviors for these patients. Physical therapy employs motor retraining to treat predominant motor symptoms (94). Finally, identifying and treating comorbid anxiety and depression remains an important consideration.

The pathophysiology of functional tremor remains unclear. Some patients have tremor that is often distractible and in some co-contraction of antagonist muscles leads to an oscillatory movement similar to clonus, with tremor resolution when the co-contraction stops (3). Many neuroimaging studies have demonstrated hypoactivation of the SMA, which is involved in movement preparation (94). Studies in functional tremor have demonstrated an increased activity of the cingulate cortex, paracingulate gyrus, and left insula compared to healthy controls (95). Neuroimaging studies have also demonstrated decreased activity of the right middle temporal gyrus, which plays a vital role in self-agency and helps to detect discrepancies between internal motor intentions and external motor actions (95). cTBS targeted at the pre-SMA has been shown to reduce abnormal sense of agency, which may be an underlying cause of FMDs in general (95). To date, no studies have implemented TMS paradigms to gain insight specifically into the pathophysiology of functional tremor, and this is an important area for future study.

### Therapeutic Use of rTMS

TMS has been used in several small, open-label studies for patients presenting with functional paresis, aphonia, mixed phenomenology (including myoclonus, Parkinsonism, and dystonia), and tremor (93). TMS paradigms used in these studies have been highly variable, making a comparison across studies difficult. Many studies have found promising results, with some reporting sustained benefit at long-term follow-up; however there is inadequate quality of evidence as there is considerable heterogeneity in the population sampled, study design, TMS parameters, and outcome measures. These studies have not included a sham arm (93). In an open-label study (*n* = 24) of patients with FMD, a single 50 pulse session of 0.25 Hz rTMS was applied over the M1 contralateral to the affected limb and the clinical scores improved by 50–75% for almost 2 years (96). Another study in patients with FMD (*n* = 33) involved a crossover design of a single session of rTMS over the contralateral motor cortex and ipsilateral spinal roots (97). There were clinical improvements in both groups, suggesting that the effects of rTMS were more cognitive-behavioral, as opposed to true neuromodulation. Given the short washout period between stimulation for the two groups, a definitive conclusion could not be drawn (97). One study employed suggestibility in their treatment design (98). Participants were told there would be a high likelihood of benefit following 5 consecutive days of a single rTMS session delivered at 0.33 Hz. The study found rTMS to premotor cortex led to improvement in physical quality of life, but there was a reduction in the psychological quality of life. These dissociative findings were attributed to the complex pathophysiology of the FMDs (98).

Two additional studies specifically focused on the response of functional tremor to rTMS. An early open-label study implemented a single session of 0.2 Hz rTMS applied to the motor cortex that led to clinical improvements, but the benefits in many patients were transient (52). There was no sham arm to rule out a placebo effect. More recently, in a randomized, double blind, active vs. sham arm study, 1 Hz rTMS at 90% RMT was delivered to the motor cortex in patients presenting with functional tremor. The study found significant and sustained clinical improvements in the active stimulation group lasting for 12 months (53). These preliminary studies suggest that rTMS can improve functional tremor; however, future studies should target specific and individualized sites determined to be hypoactive or hyperactive with fMRI and measure brain functions with TMS to characterize the pathophysiological underpinnings of functional tremor.

## Current Limitations

Although TMS can be an important tool for understanding physiology and potentially treating clinical symptoms of tremor, there are several limitations to consider. There is high variability in the rTMS paradigms and study designs used to investigate tremor syndromes, including differences in sham application, washout periods in crossover designs, target location, number of pulses, number of stimulation sessions, and duration of the stimulation in total. Many studies implemented only a single stimulation session (26, 28, 36, 37, 39, 43, 44, 51, 52, 64, 73), whereas other studies included multiple sessions ranging anywhere from 5 consecutive days to 15 stimulation days (38, 40–42, 47, 49, 50), or more unique protocols in which 5 consecutive days of stimulation are followed with a weekly session of stimulation for 3 weeks (53). The number of stimulation sessions may play a role in the duration of benefit, and thus it is critical to not only assess benefit following stimulation but also to assess how long that benefit lasts. Protocols with fewer or only single stimulation sessions would be expected to have theoretically shorter-lasting benefits than paradigms that include multiple stimulation sessions. For example, one ET study found a significant improvement in tremor scores 5 min after stimulation, but this benefit was not seen 60 min following stimulation (36). In contrast, a study incorporating 5 days of stimulation found a sustained benefit in tremor scores up to 3 weeks following stimulation (38). The number of stimulation sessions is not the only TMS parameter that may influence the duration of benefit. In fact, it may be the total number of pulses delivered to the brain that has a bigger influence on the duration of benefit as opposed to the number of stimulation sessions. Indeed, number of stimulation sessions does not appear to be linearly related to improvement in tremor, suggesting that there may be a certain threshold of pulses or sessions after which optimal clinical improvement is seen (54). In addition, the stimulation intensity, typically reported as a percentage of RMT or AMT, as well as the rate of delivered pulses may influence the degree and duration of benefit. Given the heterogeneity of rTMS paradigms between studies, it is difficult to compare and contrast results from one study to another and the optimal stimulation parameters are not yet known.

Given that the pathophysiology of each of these tremor syndromes is different from one another, the ways in which they are treated is also different. This is reflected by the current standard of care treatments, which range from specific pharmacotherapy to specific DBS target sites based on the type of tremor syndrome. Therefore, it makes sense that different TMS stimulation parameters and target sites also be needed to have the greatest therapeutic benefit for each individual tremor syndrome. However, there is variability in the TMS study designs within the same type of tremor syndrome as well. Some studies chose to randomize two separate groups of patients in an active and sham protocol (40, 41, 43, 53, 73), whereas others used a crossover design to evaluate benefit (36, 37, 39, 42, 44, 47, 51, 64). There are a few limitations specific to crossover designs performed in rTMS studies. First, a real TMS coil emits a loud clicking noise with each pulse and also generates a tapping sensation along the patient's skull throughout the procedure. Therefore, it is important to have a sham condition which mimics this active condition as closely as possible. However, sham conditions are highly variable in these studies. These sham conditions include tilting an active coil away from the target (36, 41–43, 47, 48), delivering the stimulation at a lower intensity than would be expected to cause neuromodulatory changes (28, 37, 44, 73), stimulation of neck muscles instead of the cortex (39, 64), or using a sham coil that delivers a tapping sensation accompanied by a loud clicking noise without delivering any stimulation (40, 45, 51, 53). A sham coil offers the most reliable way of ensuring blinding without unintentional neurostimulation.

A second important design factor in rTMS crossover studies is the amount of time dedicated to “washout” between the active and sham stimulation sessions. This washout period varies widely between studies, with some waiting 1–2 days (48, 51), 1 week (28, 36, 37, 39, 44, 64), or weeks to months (42, 47). It is critical to choose a washout period that will allow for stimulation effects to wear off before starting the next session. Given that paradigms with a higher total number of sessions and the total number of pulses have led to cumulative effects or longer-lasting benefits, studies implementing these paradigms should include more extended washout periods between active and sham stimulation sessions. In addition, implementing several follow-up periods, ranging from immediately after rTMS, to hours after rTMS, to weeks after rTMS, will give us a better understanding of how long we should expect different rTMS paradigm benefits to last.

These limitations, some of which are inherent to rTMS study design, make it difficult to draw any overall conclusions about the efficacy of rTMS in tremor syndromes at this time. The majority of these studies have looked at small and often heterogenous populations. Given these known limitations of rTMS studies, it is important to design future studies that will more systematically assess the therapeutic use of rTMS for tremor syndromes.

## Conclusion and Future Directions

In summary, TMS is a valuable tool that can potentially enhance the pathophysiological understanding of movement disorders. Although tremor is the most common movement disorder and recent imaging studies have advanced the knowledge of the critical pathogenic networks, TMS has been underutilized overall. Single pulse TMS paradigms have been helpful in demonstrating the brain circuitry that is likely involved in the generation of tremor. Single pulse TMS targeted to the primary motor cortex has resulted in tremor reset for both ET and rest tremor in PD (13, 14, 20, 27, 29) as well as for re-emergent tremor in PD (34), suggesting similar circuitry involved in the generation of these two tremor syndromes. However, single pulse TMS directed at the cerebellum led to tremor reset in postural tremor in PD but not in rest tremor in PD or in essential tremor, suggesting that these tremor syndromes have different underlying pathophysiology or are modified by additional factors outside of these circuits (20, 27, 29). Similarly, single pulse TMS to M1 led to reset of both ET and DTS, whereas single pulse TMS to the cerebellum led to reset in ET more so than in DTS (31). These differences suggest that certain parts of the brain circuit are more involved with the generation of tremor whereas other parts of the circuit are more involved with modulation of tremor. In addition, paired pulse TMS paradigms have demonstrated involvement of the cerebellum in ET, postural tremor of PD, and OT. TMS studies evaluating the pathophysiology of functional tremor are still needed. These pathophysiological insights are not only important for our understanding of tremor syndrome symptoms, but can also guide us into selecting appropriate rTMS parameters for treating these tremor syndromes clinically. Using knowledge of tremor pathophysiology to design rTMS studies is one important way of being more thoughtful when approaching the rTMS design for tremor syndrome studies. For example, using associative dual site TMS targeted at the M1 and dPMC was based on the assumption that each of these target sites was connected *via* different tracts to the subthalamic nucleus (STN), which is an important structure in the manifestation of tremor in PD (44). Therefore, simultaneous stimulation of these targets was hypothesized to lead to decoupling of the pathogenic oscillatory activity (44). Future pathophysiologic studies should focus on determining which brain circuits are the primary oscillator and which are more responsible for modulating existing tremor. Studies combining TMS with EEG and fMRI will be critical to answering these questions.

**Figure 3:**
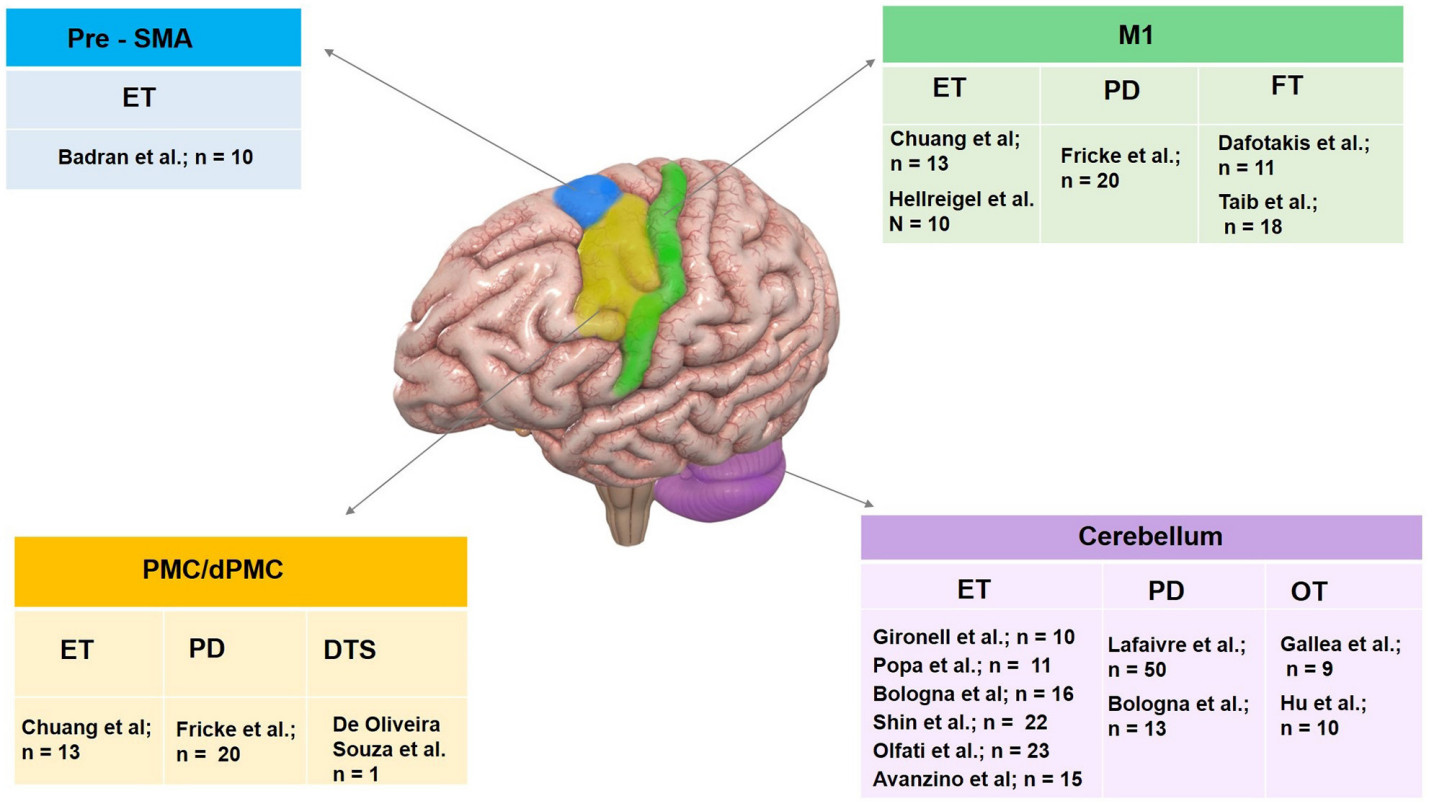
Summary of therapeutic use of rTMS in tremor syndromes organized by target site. Pre-SMA, pre supplementary motor area; M1, primary motor cortex; PMC, premotor cortex and cerebellum have been targeted. Investigator group and sample size enrolled for individual tremor syndrome including essential tremor (ET), Parkinson's disease (PD) tremor, dystonic tremor syndrome (DTS), orthostatic tremor (OT) and functional tremor (FT) are illustrated.

There is emerging evidence supporting the therapeutic potential of rTMS for treating tremor syndromes. rTMS paradigms inhibiting the cerebellum have shown promise at reducing ET, OT, and specific subtypes of PD tremor. Inhibitory paradigms targeted to the motor cortex, pre-SMA, or SMA have shown improvements in ET populations and those presenting with functional tremor (Figure 3). Although these early results are encouraging, studies involving multiple sessions, larger samples, blinded outcome assessments, and long-term follow-ups are warranted to confirm the therapeutic role of rTMS in tremors. It is crucial to include a sham-controlled arm to ensure that a placebo response does not drive clinical benefits. Future rTMS study designs would benefit from using both clinical scales and kinematic outcomes and correlating tremor improvement with underlying changes in the pathophysiology. In addition, it will be beneficial to determine if rTMS and standard pharmacological treatments have synergistic benefits. Future studies should combine TMS with imaging to identify individualized brain abnormalities and employ personalized rTMS protocols to provide robust, long-lasting benefits.

## Author Contributions

JF contributed to the writing of the first draft, conceptualization of the topic, data collection, and major revisions. CH was responsible for major revisions. MW and LK were responsible for data collection, figure illustration, and major revisions. AW was responsible for conceptualization of the topic and major revisions. All authors agree to be accountable for the content of the work.

## Conflict of Interest

The authors declare that the research was conducted in the absence of any commercial or financial relationships that could be construed as a potential conflict of interest.

## Publisher's Note

All claims expressed in this article are solely those of the authors and do not necessarily represent those of their affiliated organizations, or those of the publisher, the editors and the reviewers. Any product that may be evaluated in this article, or claim that may be made by its manufacturer, is not guaranteed or endorsed by the publisher.
